# Effects of second-line anti-tuberculosis drugs on the intestinal microbiota of patients with rifampicin-resistant tuberculosis

**DOI:** 10.3389/fcimb.2023.1127916

**Published:** 2023-04-28

**Authors:** Chunli Wu, Hengzhong Yi, Yanmei Hu, Danlin Luo, Zhigang Tang, Xinmin Wen, Yong Zhang, Mi Tang, Lizhi Zhang, Shu Wu, Mengshi Chen

**Affiliations:** ^1^ Hunan Provincial Key Laboratory of Clinical Epidemiology, Xiangya School of Public Health, Central South University, Changsha, Hunan, China; ^2^ 6th Medical Department, Hunan Province Chest Hospital, Changsha, Hunan, China; ^3^ Orthopedics and integration Medical Department, Hunan Province Chest Hospital, Changsha, Hunan, China

**Keywords:** rifampicin-resistant tuberculosis, second-line anti-tuberculosis drugs, intestinal microbiota, differential species, differential function

## Abstract

**Objective:**

To determine the effects of second-line anti-tuberculosis (TB) drugs on the composition and functions of intestinal microbiota in patients with rifampicin-resistant TB (RR-TB).

**Methods:**

In this cross-sectional study, stool samples and relevant clinical information were collected from patients with RR-TB admitted to the Drug-resistant Specialty Department at Hunan Chest Hospital (Hunan Institute For Tuberculosis Control). The composition and functions of intestinal microbiota were analyzed using metagenomic sequencing and bioinformatics methods.

**Results:**

Altered structural composition of the intestinal microbiota was found when patients from the control, intensive phase treatment, and continuation phase treatment groups were compared (P<0.05). Second-line anti-TB treatment resulted in a decrease in the relative abundance of species, such as *Prevotella copri*, compared with control treatment. However, the relative abundance of *Escherichia coli*, *Salmonella enterica*, and 11 other conditionally pathogenic species increased significantly in the intensive phase treatment group. Based on differential functional analysis, some metabolism-related functions, such as the biosynthesises of phenylalanine, tyrosine, and tryptophan, were significantly inhibited during second-line anti-TB drug treatment, while other functions, such as phenylalanine metabolism, were significantly promoted during the intensive phase of treatment.

**Conclusion:**

Second-line anti-TB drug treatment caused changes in the structural composition of the intestinal microbiota in patients with RR-TB. In particular, this treatment induced a significant increase in the relative abundance of 11 conditionally pathogenic species, including *Escherichia coli.* Functional analysis revealed significantly decreased biosynthesises of phenylalanine, tyrosine, and tryptophan and significantly increased phenylalanine metabolism.

## 1 Introduction

Tuberculosis (TB) is a chronic infectious disease that poses a threat to human health. Rifampicin-resistant TB (RR-TB) refers to the type of TB wherein the infected *Mycobacterium tuberculosis* (MTB) is confirmed to be resistant to rifampicin in any form, such as mono-resistant, poly-resistant, multidrug-resistant (MDR), and extensive drug-resistant (ER), based on *in vitro* drug sensitivity testing, regardless of whether MTB is resistant to other anti-TB drugs (Chinese Medical Association, Branch of Tuberculosis, 2019; World Health Organization, 2022). Treatment of TB becomes relatively more difficult once drug resistance develops, resulting in corresponding longer treatment durations and higher medical costs (World Health Organization, 2020; Boratibek and Manai, 2016; Dheda et al., 2019). According to the Global Tuberculosis Report 2022 published by the World Health Organization (WHO) (Organization WH., 2022), in 2021, there were 450,000 new cases of RR-TB globally, and 33,000 new cases of MDR-TB/RR-TB in China.

The intestinal microbiome is crucial in maintaining human health. Based on current research, the composition and metabolic activity of the intestinal microbiome can influence the development and function of the human immune system and play a regulatory role in the immune response against TB (Honda and Littman, 2016; Hu Y. et al., 2019). Intestinal microbiota-related disorders could not only decrease the production of mucosal-associated invariant T (MAIT) cells but also reduce the ability of these cells to produce IL-17A, thereby affecting the clearance of MTB by the body (Domingo-Gonzalez et al., 2017; Dumas et al., 2018). Short-chain fatty acids (SCFA), a type of metabolites of the intestinal microbiota, have been demonstrated to affect the body’s anti-TB immune response (Wu et al., 2020). Indole propionic acid (IPA) could inhibit the synthesis of tryptophan by MTB, thereby inhibiting MTB growth (Negatu et al., 2019). In the wake of recently emerging literature, anti-TB drugs can cause dysbiosis and alter the composition and functions of the intestinal microbiota (Wipperman et al., 2017; Hu Y. et al., 2019; Wang et al., 2020; Cao et al., 2021). In particular, the requirement for the long-term combined application of multiple second-line anti-TB drugs in patients with RR-TB has an even greater impact on the homeostasis of their intestinal microbiome (Khan et al., 2019; Eribo et al., 2020).

Anti-TB drugs are mainly divided into first-line drugs, including rifampicin (R), isoniazid (H), ethambutol (E), pyrazinamide (Z), and streptomycin, and second-line drugs, including bedaquiline (Bdq), linezolid (Lzd), moxifloxacin (Mfx), levofloxacin (Lfx), clofazimine (Cfz), cycloserine (Cs), para-aminosalicylic acid (PAS), propylthiouracil, and amikacin (Am) (Osei Sekyere et al., 2020). The treatment duration for RR-TB typically spans 18–20 months (Osei Sekyere et al., 2020), including a 6-month intensive phase and a 12–14-month continuation phase. The treatment strategy is usually customized according to the patient’s drug resistance status (Millard et al., 2015; Chou and Wenjuan, 2021). At present, studies on the impact of anti-TB treatment on patients’ intestinal microbiota have focused more on a standard regimen comprising first-line drugs, including H, R, Z, and E. According to existing studies, HRZE (isoniazid + rifampin + pyrazinamide + ethambutol) treatment could cause significant changes in the composition and metabolic functions of patients’s intestinal microbiota, which persist after completion of TB treatment for more than 1 year (Eribo et al., 2020). However, little attention has been paid to the effects of second-line anti-TB drugs on the intestinal microbiota. Compared with 16S DNA sequencing used in previous studies, metagenomic analysis provides direct access to nucleic acid information in stool samples, enabling a more truthful reflection of the intestinal microbial composition in samples and exploration of the metabolic pathways and genetic functions of the intestinal microbiome at the molecular level (Chen and Pachter, 2005; Tringe et al., 2005).

In this study, we used metagenomics to analyze the differences in intestinal microbiota among patients with RR-TB who were on second-line anti-TB drugs during the intensive and continuation phases and patients who were solely on conventional first-line anti-TB drugs and to assess the microbial composition and differential strains in patients on second-line anti-TB drugs during the intensive and continuation phases. Functional analysis was subsequently conducted to explore the alteration in related intestinal microbiota functions.

## 2 Materials and methods

### 2.1 Study design and population

In this cross-sectional study, stool samples and clinical data were collected from patients with RR-TB admitted to the Drug-resistant Specialty Department at Hunan Chest Hospital (Hunan Institute For Tuberculosis Control) from September 2021 to May 2022. The inclusion criteria were as follows: patients aged >18 years; patients that understood the study design and participated voluntarily in the study; patients who were infected with MTB and confirmed to be at least resistant to rifampicin by conventional phenotypic testing/genetic testing (People's Republic of China state health and Family Planning Commission, 2018; World Health Organization, 2020). The exclusion criteria were as follows: patients with intestinal TB; patients with combined heart, brain, kidney, liver, or other organ dysfunctions; patients with combined gastrointestinal diseases; and patients who were intolerant to second-line anti-TB drug treatment. All protocols were approved by the Medical Ethics Committee of Hunan Chest Hospital (LS2020111101).

In this study, the treatment regimen for RR-TB was established mainly based on the “Chinese expert consensus on multidrug-resistant tuberculosis and Rifampicin-resistant tuberculosis treatment (2019)” developed by the Tuberculosis Branch of Chinese Medical Association (Chinese Medical Association, Branch of Tuberculosis, 2019). The specific strategies are outlined below: apply all Group A anti-TB drugs combined with at least one Group B anti-TB drug; when only 1-2 Group A anti-TB drugs are applicable, all Group B anti-TB drugs should be used; when a proper regimen cannot be developed by selecting Group A and Group B anti-TB drugs, Group C anti-TB drugs should be considered, and the patient’s past drug history and drug sensitivity test results should be taken into account. The groups were as follows: Group A: drugs of choice, including Lfx or Mfx, Bdq, and Lzd; Group B: drugs of second choice, including Cfz and Cs; and Group C: alternative drugs, including Z, E, delamanid (Dlm), protionamid (Pto), Am or capreomycin (Cm), PAS, imipenem/cilastatin (Ipm/Cln), or meropenem (Mpm) in order of preference.

### 2.2 Stool samples and general demographic information collection

Approximately 10 g of fresh stool sample was collected from each patient using sterile collection tubes, transported at low temperature to the laboratory, marked with the identification number of the study participants, and stored immediately at -80°C. Demographic information, including the patient’s age, gender, height, weight, history of diseases, treatment duration for drug-resistant TB, and treatment regimen, was collected from the hospital case management system.

### 2.3 Metagenomic sequencing

Metagenomic sequencing was performed by Novogene Co., Ltd (Beijing, China). The detailed procedures were as follows: microbial DNA was extracted from the stool samples using the magnetic beads-based kit (Tiangen, Beijing, China). DNA purity and integrity were evaluated *via* 1% agarose gel electrophoresis (AGE), and DNA quantity was measured using the Qubit^®^ dsDNA Assay Kit in Qubit^®^ 2.0 Fluorometer (Life Technologies, CA, USA). An appropriate amount of sample was transferred into a centrifuge tube and diluted with sterile water to achieve an OD value of 1.8–2.0. The library was constructed with 1 μg of the genomic DNA using the NEBNext^®^ Ultra DNA Library Prep Kit for Illumina (NEB, USA). The sample was then divided into random ~350 bp fragments using a Covaris ultrasonic fragmentation machine. The steps of end repair, poly-A tail addition, adapters addition, purification, and PCR amplification were performed to complete the preparation of the entire genomic library. After the library was constructed, Qubit 2.0 was used for initial quantification. The library was diluted to 2 ng/uL and the insert size was detected using Agilent 2100. Once the insert size met the expectation, the effective concentration of the library was accurately measured using Q-PCR to ensure its quality (effective library concentration > 3 nM). After the library passed the quality test, the different libraries were pooled according to the effective concentration and the target data input volume, and then sequenced using Illumina PE150 to obtain the raw data.

### 2.4 Metagenomic analysis

#### 2.4.1 Analysis and annotation of metagenomic sequences

(1) Pre-processing of sequencing data

Clean data were obtained after quality control (QC) of raw data with Readfq (V8) and filtration of the reads that may have originated from the host with Bowtie2 software (version 2.2.4).

(2) Metagenome assemblies

Clean data were assembled and analyzed using the MEGAHIT software (v1.0.4-beta).

(3) Gene prediction and abundance analysis

MetaGeneMark (V3.05) was used for gene prediction of the assembled scaftigs, and CD-HIT software (V4.5.8) was used to remove redundancy to obtain a non-redundant initial gene catalogue. The final gene catalogue (Unigenes) for subsequent analysis was obtained using Bowtie2 (Bowtie2.2.4) to compare the Clean Data of each sample to the initial gene catalogue, and the abundance of each gene in each sample was calculated. Based on the abundance information on each gene in Unigenes in each sample, basic information was collected and core-pan genome analysis was performed.

(4) Species and function annotations

Species annotation was performed by comparing Unigenes to the sequences of bacteria, fungi, archaea, and viruses retrieved from NCBI’s NR database (Version 2018-01-02) using DIAMOND software (V0.9.9.110). The LCA algorithm (applied in the systematic classification of MEGAN software) was adopted to determine the information of species annotation.

Functional annotation was achieved by comparing Unigenes with the KEGG database (Kanehisa et al., 2014) (Version 2018-01-01) using DIAMOND software (vO.9.9.110), and then analyzing the Best Blast Hit results to tabulate the relative abundances of functions at different functional levels.

#### 2.4.2 Analysis of species and functional composition

Starting from the abundance tables at each taxonomic level, the species and functions that had the top 10 relative abundance in each level were presented in distribution histograms to visualize the details and differences in their compositions.

#### 2.4.3 Diversity analysis

(1) Alpha diversity analysis: The Richness, Shannon, Simpson, Pielou, invsimpson, Chao1, ACE, and goods coverage index were used to reflect the diversity of the microbiota in the samples. Alpha diversity analysis was performed on Tutools platform (https://www.cloudtutu.com), a free online data analysis website.

(2) Beta diversity analysis: Principal Coordinate Analysis (PCoA) is a visualization method for studying similarities or differences of data. Non-metric multidimensional scaling (NMDS) analysis is often used to compare the inter-group differences of samples. With each point representing a sample and the points of the same color coming from the same group, a smaller distance between two points indicates a smaller difference in their microbiota composition. PCoA (R ade4 package, Version 2.15.3) and NMDS analysis (R vegan package, Version2.15.3) were performed, followed by Anosim analysis (R vegan package, Version 2.15.3) to determine the differences between groups.

#### 2.4.4 Analysis of inter-group differences

Based on the relative abundance tables for species and functions, the species or functions present in at least 10% of the samples were selected and analyzed for inter-group differences using LEfSe software (Default Linear Discriminant Analysis (LDA) Score of 3).

### 2.5 Statistical analysis

Excel 2019 was used to organize the case information, and SPSS 23.0 was used for statistical description and inference. Statistical description: quantitative data that conformed to normal distribution are presented as mean ± SD, and quantitative data that did not conform to normal distribution are presented as median (M) and interquartile range; qualitative data are presented as the number of cases and proportion. Statistical inference: Analysis of variance (ANOVA) was used for quantitative data that conformed to the normal distribution and a non-parametric test was used for quantitative data that did not conform to normal distribution; qualitative data were tested using the McNemar chi-square test when conformed to normal distribution and Fisher’s exact test when the data did not conform to normal distribution.

## 3 Results

### 3.1 General demographic characteristics

A total of 150 patients with RR-TB were included in this study: 30 patients who were initially diagnosed with RR-TB but did not receive second-line drug therapy (control group), 60 patients who were at the 6-month intensive phase of the treatment with second-line anti-TB drugs (G1G2 group), and 60 patients who were at the 12–14-month continuation phase of the treatment with second-line anti-TB drugs (G3G4 group). The demographic characteristics of patients in the control, G1G2, and G3G4 groups are shown in **Table 1**. Based on statistical analysis, no significant differences were noted in age, gender, and body mass index (BMI) among the three groups. Meanwhile, the G1G2 group received second-line anti-TB drugs for an average of 87 days, while the G3G4 group received second-line anti-TB drugs for an average of 373 days. On examining information on the main second-line anti-TB drugs used in the study population, no significant difference was noted in the use of Mfx, Lfx, Cs, PAS, Pto, and Am between the G1G2 and G3G4 groups. However, a greater use of three drugs, i.e. Bdq, Lzd, and Cfz, was recorded for the G1G2 group. The details are provided in **Table 2**.

**Table 1:** Demographic characteristics of the participants (n = 150).

Variables	Control (n=30)	G1G2 (n=60)	G3G4 (n=60)
Age (Mean ± SD)	40.27 ± 17.75	46.63 ± 15.26	44.75 ± 15.83
Female (%)	13 (43.3%)	17 (28.3%)	16 (26.7%)
Body Mass Index (BMI)	20.57 ± 3.01	21.73 ± 3.17	20.81 ± 3.04
Time on TB treatment (Days)	–	87.47 ± 41.26	373 ± 151.37

**Table 2:** Second-line anti-tuberculosis drug use in patients in the intensive and consolidation treatment groups.

Type of drug	G1G2 (n=60)	G3G4 (n=60)	*χ^2^*	*P*
Bdq ^*^			4.910	0.027
Yes	18 (30.0%)	8 (13.3%)		
No	42 (70.0%)	52 (86.7%)		
Lzd ^*^			5.263	0.022
Yes	50 (83.3%)	39 (65.0%)		
No	10 (16.7%)	21 (35%)		
Mfx			3.001	0.083
Yes	35 (58.3%)	44 (73.3%)		
No	25 (41.7%)	16 (26.7%)		
Lfx			3.333	0.068
Yes	16 (26.7%)	8 (13.3%)		
No	44 (73.3%)	52 (86.7%)		
Cfz ^*^			5.167	0.023
Yes	28 (46.7%)	16 (26.7%)		
No	32 (53.3%)	44 (73.3%)		
Cs			0.288	0.591
Yes	53 (88.3%)	51 (85.0%)		
No	7 (11.7%)	9 (15.0%)		
PAS			0.352	0.553
Yes	20 (33.3%)	17 (28.3%)		
No	40 (66.7%)	43 (71.7%)		
Pto			2.143	0.143
Yes	24 (40.0%)	32 (53.3%)		
No	36 (60.0%)	28 (46.7%)		
Am			1.205	0.272
Yes	6 (10.0%)	2 (3.3%)		
No	54 (90.0%)	58 (96.7%)		

^*^P < 0.05, There was a significant difference between the G1G2 group and G3G4 group.

### 3.2 Second-line anti-TB drugs induce compositional changes in the intestinal microbiota species of patients with RR-TB without affecting intestinal microbiota diversity

**Figure 1:**
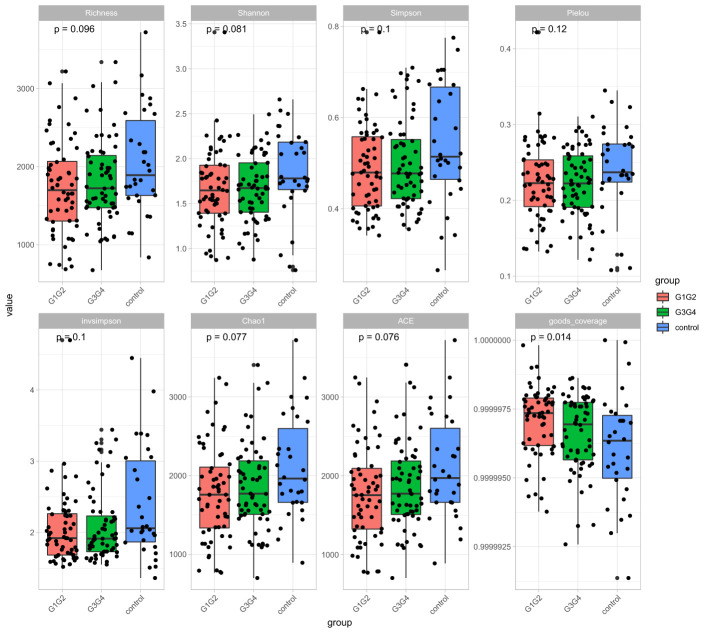
Alpha diversity indices included Richness, Shannon, Simpson, Pielou, invsimpson, Chao1, ACE, and goods coverage. The Alpha diversity index was plotted as a cumulative box plot of species to assess differences in species richness and diversity of microbial communities among the control, G1G2 and G3G4 groups.

**Figure 2:**
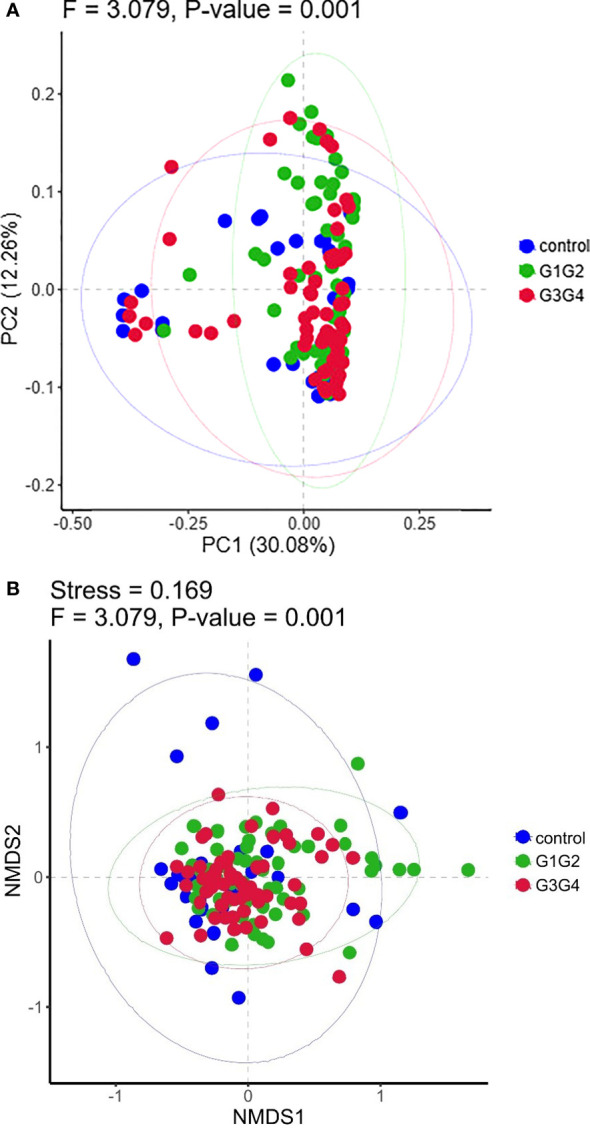
**(A)** Principal Coordinates Analysis (PCoA) plot based on Bray-Curtis distance. Each dot represents one sample from each group and samples from the same group are represented using the same color. **(B)** The non-metric multidimensional scaling (NMDS) analysis was used to compare the differences between the groups in the sample. Each dot represents one sample from each group, the distance between dot indicates the degree of variation and samples from the same group are represented using the same color.

No significant differences were found in the Shannon index or Simpson index (**Figure 1**) among the three groups (all *P* > 0.05), indicating that the application of second-line anti-TB drugs in patients with RR-TB in this study did not result in any significant changes in the diversity of intestinal microbiota. This finding may be related to our selection of patients who were initially diagnosed with RR-TB and received conventional first-line anti-TB treatment but not second-line drugs, as the controls. Beta diversity analysis was performed using PCoA and NMDS analysis. The microbiota distributions of different samples in the G1G2, G3G4, and control groups differed in two dimensions, and the species composition of the G1G2 group had few similarities to species composition of the control group. In contrast, the species composition of the G3G4 group was more similar to that of the control and G1G2 groups, as shown in **Figures 2A, B**. The differences among the three groups were statistically significant (all *P* < 0.05) in the Adonis test, as shown in **Table 3**. Therefore, compared with patients who were initially diagnosed with RR-TB and had not received treatment with the second-line anti-TB drug, patients with RR-TB and administered second-line anti-TB drugs displayed changes in the species composition of the intestinal microbiota, with the second-line anti-TB drugs applied during the intensive phase resulting in greater changes without affecting the microbiota diversity.

**Table 3:** Adonis test for differences between groups.

groups	*F*	*P*
Control vs G1G2	4.9322	0.001
G1G2 vs G3G4	2.2168	0.017
Control vs G3G4	2.2941	0.035

### 3.3 Effects of second-line anti-TB drug treatment duration on the relative abundance of intestinal microbiota in patients

**Figure 3:**
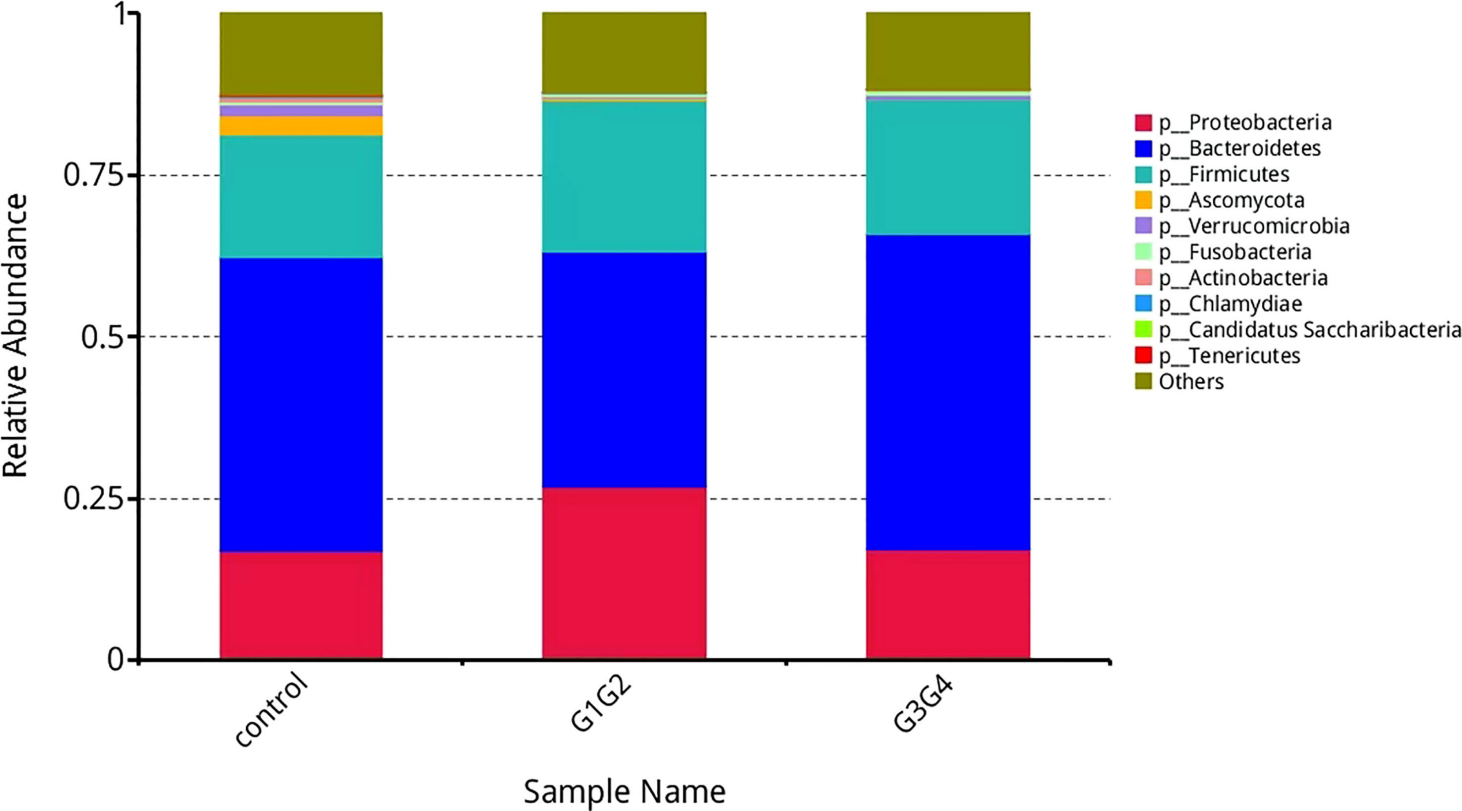
Barplot showing the composition of the top 10 microorganisms in terms of maximum relative abundance at the phylum level.

As shown in **Figure 3**, at the phylum level, the dominant species we observed to dominate were mainly: *Bacteroidetes*, *Proteobacteria*, and *Firmicutes*. Compared to the control group (16.8%), the relative abundance of *Proteobacteria* showed a significant increase (*P* < 0.05) in the G1G2 group (26.8%) and a significant decrease (*P* < 0.05) in the G3G4 group (17.2%). The relative abundance of *Firmicutes* showed an increase (*P* > 0.05) in the G1G2 group (23.6%) and a decrease (*P* > 0.05) in the G3G4 group (20.8%) compared to the control group (18.9%). The relative abundance of *Bacteroidetes* showed a decrease (*P* > 0.05) in the G1G2 group (36.2%) and a significant increase (*P* < 0.05) in the G3G4 group (48.8%) compared to the control group (45.6%). Detailed *P* values are shown in **Tables 4**, **5**.

**Table 4:** Comparison of the top ten gut microbiota in relative abundance in the control and G1G2 groups at the phylum level.

Comparison	phylum	*P* value
control - G1G2	*p:Bacteroidetes*	0.075
control - G1G2	p:Proteobacteria^*^	0.024
control - G1G2	*p:Firmicutes*	0.193
control - G1G2	*p:Ascomycota*	0.220
control - G1G2	*p:Verrucomicrobia*	0.065
control - G1G2	p:Actinobacteria^*^	0.024
control - G1G2	*p:Fusobacteria*	0.065
control - G1G2	p:Chlamydiae^*^	0.041
control - G1G2	p:Candidatus Saccharibacteria^*^	0.000
control - G1G2	*p:Tenericutes*	0.243

Wilcoxon test was used for comparison between groups and p-values were corrected using the Benjamini-Hochberg (BH) method.^*^P < 0.05, There was a significant difference between the control group and G1G2 group.

**Table 5:** Comparison of the top ten gut microbiota in relative abundance in the G1G2 and G3G4 groups at the phylum level.

Comparison	phylum	*P* value
G1G2 - G3G4	p:Bacteroidetes^*^	0.021
G1G2 - G3G4	*p:Firmicutes*	0.285
G1G2 - G3G4	p:Proteobacteria^*^	0.041
G1G2 - G3G4	*p:Verrucomicrobia*	0.597
G1G2 - G3G4	*p:Fusobacteria*	0.436
G1G2 - G3G4	*p:Actinobacteria*	0.832
G1G2 - G3G4	*p:Ascomycota*	0.072
G1G2 - G3G4	*p:Chlamydiae*	0.213
G1G2 - G3G4	*p:Tenericutes*	0.665
G1G2 - G3G4	*p:Candidatus Saccharibacteria*	0.234

Wilcoxon test was used for comparison between groups and p-values were corrected using the Benjamini-Hochberg (BH) method.^*^P < 0.05, There was a significant difference between the G1G2 group and G3G4 group.

### 3.4 Effects of second-line anti-TB drugs on patients’ intestinal microbiota

**Figure 4:**
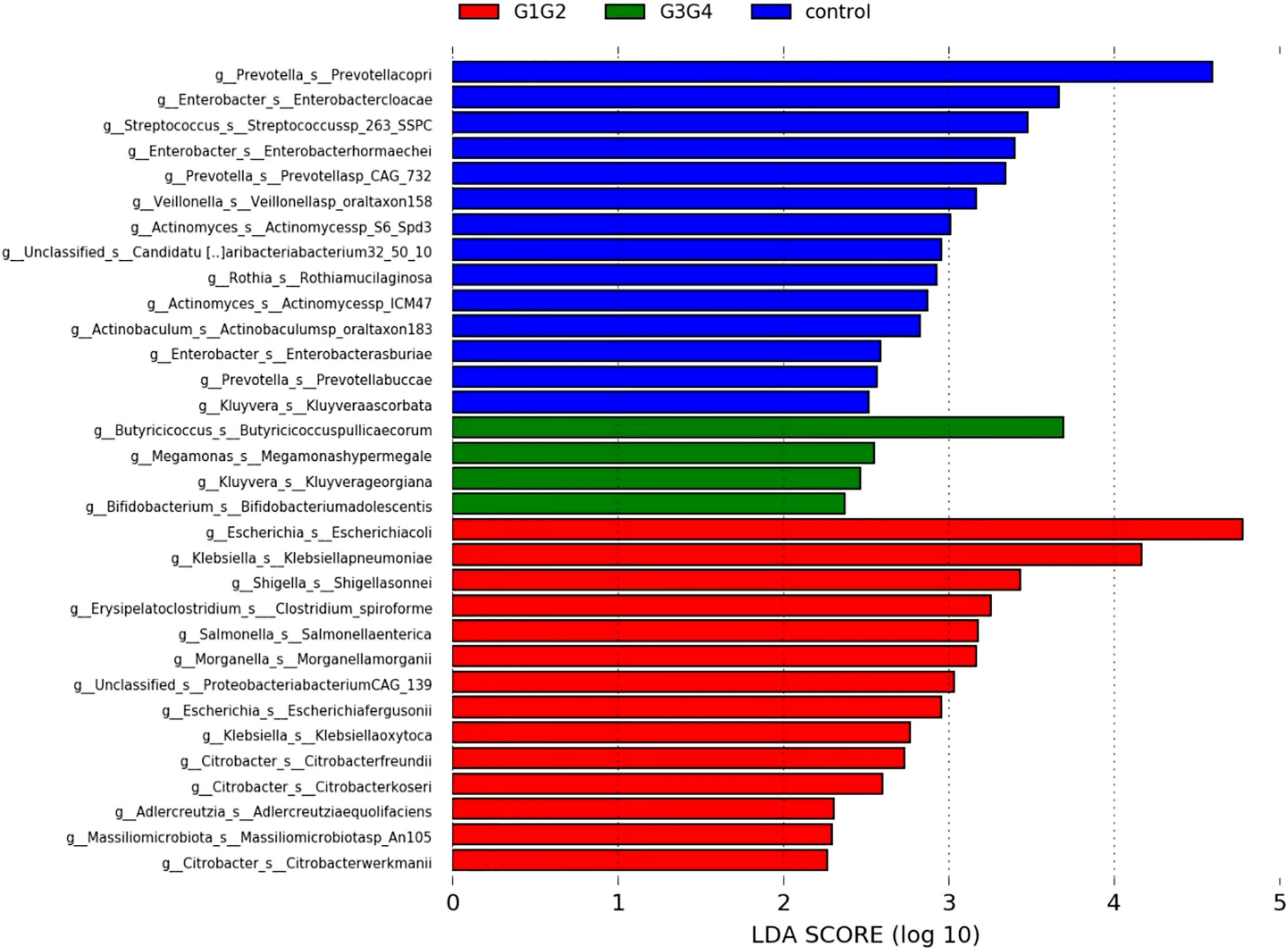
Significant differences in relative abundance identified by LEfSe analysis between *the three groups (Control, G1G2 and G3G4).* At the species level, the untreated group(control) is shown in blue, the intensive treatment group(G1G2) in red and the consolidation treatment group(G3G4) in green. The length of the column represents the influence of significantly different species in relative abundance (LDA scores > 2).

To further explore the effect of second-line anti-TB drug treatment on the intestinal microbiota, LEfSe was employed to analyze the differences between the control, G1G2, and G3G4 groups at the species level, as shown in **Figure 4**. Second-line anti-TB drug treatment resulted in a significant decrease in the relative abundances of 13 microbial species compared with that noted in the control group. Among these species, the relative abundance of *Actinomyces* sp. *ICM47*, *Actinomyces* sp. *S6-Spd3*, and *Actinobaculum* sp. *oral taxon 183* was significantly decreased in the G1G2 and G3G4 groups, while the relative abundance of *Prevotella buccae*, *Prevotella copri*, and *Prevotella* sp. *CAG: 732* decreased and then gradually increased in the G1G2 and G3G4 groups. The detailed relative abundances are shown in **Table 6**.

**Table 6:** Relative abundance of differential strains enriched in the control group.

species	Control	G1G2	G3G4
*Actinobaculum* sp. *oral taxon 183*	0.000197	0.000001	0.000008
*Actinomyces* sp. *ICM47*	0.000136	0.000000	0.000001
*Actinomyces* sp. *S6-Spd3*	0.00093	0.000000	0.000004
*Enterobacter asburiae*	0.000103	0.000054	0.000055
*Enterobacter cloacae*	0.000033	0.000006	0.000022
*Enterobacter hormaechei*	0.00062	0.000218	0.000111
*Prevotella buccae*	0.000323	0.000023	0.000133
*Prevotella copri*	0.043054	0.004963	0.030959
*Prevotella* sp. *CAG:732*	0.002427	0.000259	0.001102
*Rothia mucilaginosa*	0.000166	0.000000	0.000006
*Streptococcus* sp. *263_SSPC*	0.001447	0.000017	0.000032
*Candidatus Saccharibacteria bacterium 32-50-10*	0.000088	0.000000	0.000000
*Veillonella* sp. *oral taxon 158*	0.000731	0.000025	0.000086

There were 14 differentially enriched species in the intensive phase treatment group: *Proteobacteria*, *Citrobacter freundii*, *Citrobacter koseri*, *Citrobacter werkmanii*, *Escherichia coli*, *Escherichia fergusonii*, *Klebsiella oxytoca*, *Klebsiella pneumoniae*, *Morganella morganii*, *Salmonella enterica*, *Shigella sonnei*, *Proteobacteria bacterium CAG:139*, and *Clostridium spiroforme*. The detailed relative abundances of these species are presented in **Table 7**. In addition, compared to the control group, the relative abundances of *Bifidobacterium adolescentis* and *Megamonas hypermegale* decreased in the G1G2 group and increased significantly in the G3G4 group. The relative abundances of *Butyricicoccus pullicaecorum* and *Kluyvera georgiana* also increased significantly in the G3G4 group. The detailed relative abundances are shown in **Table 8**.

**Table 7:** Relative abundance of differential strains enriched in the G1G2 group.

species	Control	G1G2	G3G4
*Citrobacter freundii*	0.000068	0.000323	0.000133
*Citrobacter koseri*	0.00011	0.000356	0.000128
*Citrobacter werkmanii*	0.000001	0.000073	0.000024
*Escherichia coli*	0.014555	0.044835	0.032015
*Escherichia fergusonii*	0.000251	0.000712	0.000461
*Klebsiella oxytoca*	0.000179	0.000448	0.000200
*Klebsiella pneumoniae*	0.009327	0.010599	0.004077
*Morganella morganii*	0.000015	0.000687	0.000374
*Salmonella enterica*	0.00061	0.001511	0.000968
*Shigella sonnei*	0.00067	0.001999	0.001538
*Proteobacteria bacterium CAG:139*	0.000478	0.001026	0.000104
*Adlercreutzia equolifaciens*	0.000037	0.000163	0.000008
*Clostridium spiroforme*	0.00027	0.001192	0.000781
*Massiliomicrobiota* sp. *An105*	0.000006	0.000084	0.000043

**Table 8:** Relative abundance of differential strains enriched in the G3G4 group.

species	Control	G1G2	G3G4
*Bifidobacterium adolescentis*	0.000022	0.000008	0.000121
*Butyricicoccus pullicaecorum*	0.000239	0.001858	0.002950
*Kluyvera georgiana*	0.000048	0.000115	0.000228
*Megamonas hypermegale*	0.000127	0.000007	0.000220

### 3.5 Second-line anti-TB drug therapy affects the functions of the patient’s intestinal microbiota

**Figure 5:**
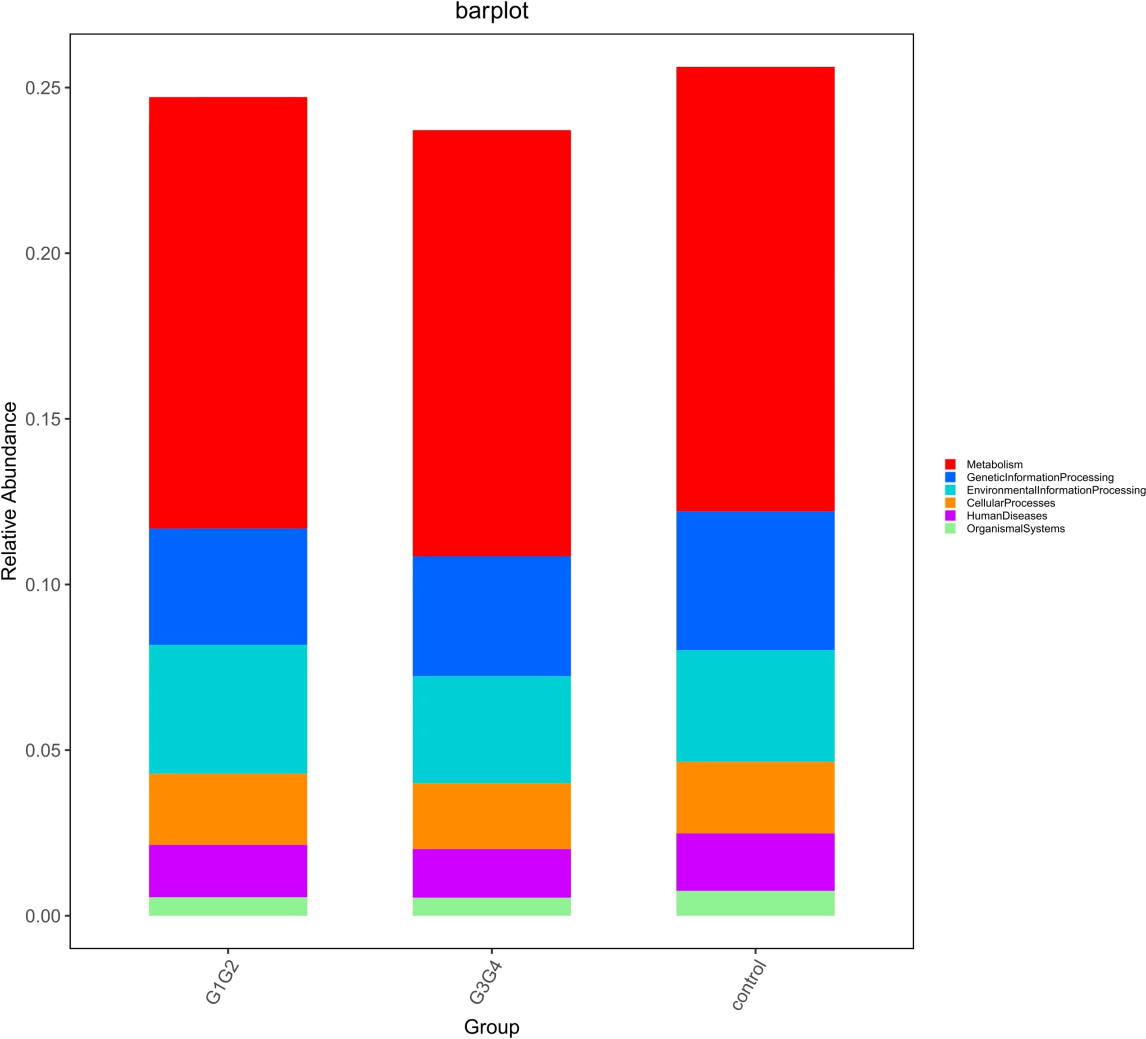
Barplot showing the composition of the top 6 functions in terms of maximum relative abundance at the KEGG level1.

**Figure 6:**
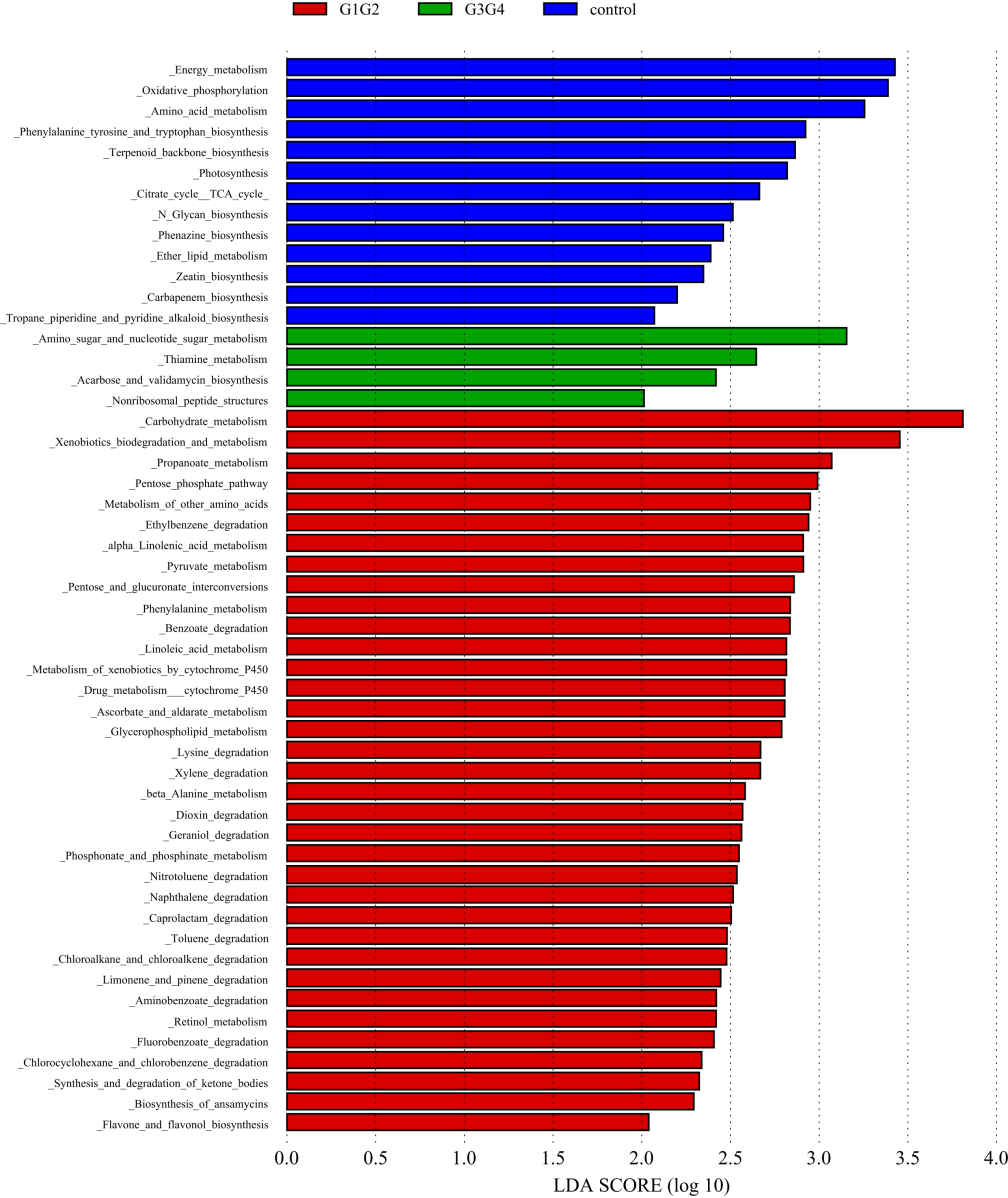
Significant differences in relative abundance identified by LEfSe analysis between the three groups (Control, G1G2 and G3G4). In the metabolism function module (level 3), the untreated group(control) is shown in blue, the intensive treatment group(G1G2) in red and the consolidation treatment group(G3G4) in green. The length of the column represents the influence of significantly different species in relative abundance (LDA scores > 2).

To explore the potential physiological functions of intestinal microbiota during second-line anti-TB drug treatment by comparing the results with the KEGG functional library. Among the 6 major functional pathways in the first level of the KEGG metabolic network, all groups had the highest relative abundances in metabolism-related functions (**Figure 5**). LEfSe analysis was used to identify functions that differed significantly between the three groups on the metabolic function module at level 3 of the KEGG pathway. As shown in **Figure 6**, the functions that were significantly enhanced in the control group were mostly related to biosynthetic functions, such as Phenylalanine, tyrosine and tryptophan, Phenazine, N-Glycan, Zeatin, Terpenoid backbone, Carbapenem and other substances of biosynthesis function.

The functions that were significantly enhanced in the G1G2 group were mostly related to the metabolism functions of Phenylalanine, Linoleic acid, beta-Alanine, Propanoate, Ascorbate and aldarate, Glycerophospholipid, etc., and the degradation functions of Lysine, Benzoate, Nitrotoluene, Aminobenzoate, Caprolactam, etc. Moreover, the functions enriched in the G1G2 group are Drug metabolism-cytochrome P450, Metabolism of xenobiotics by cytochrome P450, Pentose phosphate pathway, and Pentose and glucuronate interconversions. Acarbose and validamycin biosynthesis, Amino sugar and nucleotide sugar metabolism, and Thiamine metabolism were significantly enhanced in the G3G4 group.

## 4 Discussion

TB, a chronic infectious disease with a long treatment duration and high level of drug resistance, has had significant impacts on human health since ancient times (Dheda et al., 2019). According to current studies, the conventional HRZE anti-TB drugs could cause changes in the composition of intestinal microbiota, especially when the drugs are applied for long-term treatment. However, the specific effects of second-line anti-TB drugs on the intestinal microbiota remain unclear (Wipperman et al., 2017; Eribo et al., 2020). In addition, intestinal microbiomes are known to be associated with the body’s immunity, while the clearance of MTB in patients with TB is closely linked to patient’s immunity status. Therefore, it is extremely important to clarify how second-line anti-TB drugs induce changes in patient’s intestinal microbiota.

In this study, the intestinal microbiota diversity (Shannon and Simpson indexes) did not change significantly in patients treated with second-line anti-RR-TB drugs during the intensive and continuation phases compared with that noted in patients in the control group. However, significant differences were noted in the structural composition of their intestinal microbiota. Similar to our findings, Jinyu Wang et al. (2020) reported no significant change in the Shannon index of the intestinal microbiota in patients treated for MDR-TB. Nonetheless, a significant difference was found in the structural composition of the intestinal microbiota.

The relative abundances of *Actinobaculum* sp. *oral taxon 183*, *Actinomyces* sp. *ICM47*, and *Actinomyces* sp. *S6-Spd3* decreased rapidly in the second-line anti-TB drug-treated intensive and continuation phase groups compared with the control group, aligning with the findings of Wang J et al (Wang et al., 2020), who revealed that the relative abundance of *Actinomyces* in the MDR treatment group decreased significantly. These three species belong to the family *Actinomycetaceae*, which can influence butyrate production through the production of butyrate precursors (e.g. acetate and succinate) (Esquivel-Elizondo et al., 2017). Butyrate can promote the expression of the anti-inflammatory factor, IL-10, and inhibit the expression of pro-inflammatory factors, thereby inhibiting the phagocytic and bactericidal effects of macrophages and ultimately their ability to clear MTB (Segal et al., 2017; Serino, 2019). In contrast, compared with the control, the relative abundances of *Prevotella buccae*, *Prevotella copri*, and *Prevotella* sp. *CAG:732*, which are three species that belong to genus *Prevotella*, decreased rapidly during the intensive phase of treatment and recovered gradually during the continuation phase. A decrease in the relative abundance of *Prevotella* was found in patients treated with conventional anti-TB drugs (Wipperman et al., 2017). *Prevotella* can affect glucose metabolism by promoting the storage of glycogens, the decrease of which may lead to nutritional deficiencies and weakened body immunity, affecting the clearance of MTB (Kovatcheva-Datchary et al., 2015; Kang et al., 2021). Another study reported that *Prevotella* might modulate the body’s immunity to affect the prognosis and outcomes of patients with TB (Luo et al., 2017). *Prevotella copri* can ferment dietary fibres to produce SCFAs, such as formic acid, acetic acid, and succinic acid (De Filippis et al., 2016; Franke and Deppenmeier, 2018). These SCFAs not only have high antimicrobial activity but also promote the proliferation of type 3 innate lymphoid cells (ILC3s) (Corrêa-Oliveira et al., 2016; Chun et al., 2019). ILC3s are associated with the increase in alveolar macrophages, which can protect the host from MTB infection (Ardain et al., 2019).

Notably, the second-line anti-TB drug-treated intensive phase group was enriched with 11 conditionally pathogenic species of *Pseudomonadota*, including *Escherichia coli*, *Escherichia fergusonii*, *Klebsiella oxytoca*, *Klebsiella pneumoniae*, *Salmonella enterica*, and *Shigella sonnei* (Rajilić-Stojanović and de Vos, 2014; Rizzatti et al., 2017; Connolly et al., 2018). Among the four major phyla of the intestinal microbiota (*Bacillota*, *Bacteroidota*, *Pseudomonadota*, and *Actinomycetota*), *Pseudomonadota* is the most unstable (Faith et al., 2013). The enrichment of the *Pseudomonadota* phylum in the intestine might reflect intestinal dysbiosis (Shin et al., 2015). Similarly, the relative abundance of *Clostridium spiroforme* (belonging to *Erysipelatoclostridium*) was significantly increased in the group treated with second-line anti-TB drugs during the intensive phase, and *Erysipelatoclostridium* was significantly enriched in patients on conventional anti-TB treatment (Namasivayam et al., 2017). *Clostridium spiroforme* can produce *C. spiroforme* toxins, which cause intestinal diseases (Songer and Uzal, 2016). Second-line anti-TB drug treatment is speculated to cause intestinal dysbiosis in patients, which reportedly affected the normal proliferation of immune cells and autophagic processes, reducing the patient’s resistance to MTB (Osei Sekyere et al., 2020), thereby negatively affecting the outcome of treatment in patients with drug-resistant TB.

The relative abundance of *Bifidobacterium adolescentis* decreased in the intensive phase treatment group and increased significantly in the continuation phase treatment group. Similarly, the relative abundance of *Bifidobacterium adolescentis* decreased at 1 week of conventional anti-TB treatment and increased at the time of cure (Hu Y. et al., 2019). *Bifidobacterium* is known to be a common probiotic currently available as a probiotic preparation or for fecal microbiota transplantation (Gupta et al., 2016). *Bifidobacterium* supplements can improve lung function in elderly patients with pneumonia (Hu M. et al., 2019) and has been demonstrated to induce Th17 cell responses, which is beneficial in protecting the intestinal barrier and clearing MTB (Tan et al., 2016; Wipperman et al., 2017). *Butyricoccus pullicaecorum*, enriched during the continuation phase of the treated group, is a probiotic that produces butyrate and is essential for maintaining gastrointestinal health (Geirnaert et al., 2014). Interestingly, the relative abundance of *Megamonas hypermegale* decreased during the intensive phase of the treatment but increased significantly to a level even higher than that of the control group during the continuation phase of the treatment. Wang et al. (2020) found a significant decrease in the relative abundance of *Megamonas* after treatment for MDR, and *Megamonas* was demonstrated to be associated with the production of SCFAs (butyrate and propionate) in the human intestine (De Paepe et al., 2017).

Although the second-line anti-TB drugs used for the treatment of drug-resistant TB could effectively fight drug-resistant MTB, they have serious impacts on the functions of the intestinal microbiota. Most of the differential functions identified among the three groups were mainly found in the metabolic module. Based on the results, the biosynthetic functions of phenylalanine, tyrosine, tryptophan, phenolic acid, and N-glycans were significantly hindered during anti-RR-TB treatment, aligning with the findings of Maji A et al., who found that the anabolic functions of amino acid and cofactor were impeded significantly in patients with TB (Maji et al., 2018), while the functions of degrading substances, such as lysine and fatty acids, were significantly promoted, and the functions of metabolizing substances, such as phenylalanine, tyrosine, ascorbic acid and aldehyde, propionate, and pyruvate were significantly enhanced during the intensive phase of treatment. This result does not conflict with that of a previous study, in which the functions of metabolizing cofactors, vitamins, and lipopolysaccharide were found to be significantly enhanced after conventional anti-TB treatment (Zhang et al., 2022). Notably, the function of drug metabolism-cytochrome P450 was found to be significantly promoted during the intensive phase of treatment and decreased during the continuation phase. Cytochrome P450 could be a pharmaceutical target for the treatment of MTB infection (Ortiz de Montellano, 2018).

In summary, we applied a metagenomic sequencing approach to characterize the intestinal microbiota of patients at different stages of the second-line anti-TB drug therapy. By understanding the key gut microbial species associated with second-line anti-TB drugs treatment, we can further improve the treatment for RR-TB by reconstructing the normal intestinal microbiota of patients with RR-TB.

## 5 Limitations

This study was a cross-sectional study in which patients in the control group had already received conventional first-line anti-TB treatment at the time of initial diagnosis of RR-TB but were not on second-line drug treatment. Healthy individuals and patients with TB who had not received any anti-TB treatment were missing as controls, the translational part of the study has some limitations. Patients in this study were grouped according to the stages of their anti-drug-resistant TB treatment, a metagenomic sequencing technique was used to annotate the microbiome at the species level, and an in-depth functional analysis was conducted. Overall, the results of this study provide certain reference values.

## 6 Conclusion

In this study, second-line anti-TB drug treatment resulted in changes in the structural composition of the intestinal microbiota, with significantly increased relative abundances of 11 harmful species, such as *Escherichia coli* and *Salmonella enterica*, in patients at the intensive phase of the treatment. Meanwhile, functional analysis revealed that the anabolic functions of the patient’s intestinal microbiota, such as biosynthesises of phenylalanine, tyrosine, and tryptophan, were significantly inhibited, while the functions of degrading substances, such as lysine and fatty acids, and metabolizing substances, such as phenylalanine and tyrosine, were significantly enhanced.

## Data availability statement

The data presented in the study are deposited in the NCBI repository, accession number PRJNA932263.

## Ethics statement

The studies involving human participants were reviewed and approved by Medical Ethics Committee of Hunan Chest Hospital. The patients/participants provided their written informed consent to participate in this study.

## Author contributions

HY, CW, MC, and YH designed the idea and method of this study. DL, ZT, XW, YZ, MT, LZ, and SW organize and verify the data of this study. HY and CW analyzed the data. CW wrote the manuscript. HY, MC, and YH supervised and supported the study. All authors contributed to the article and approved the submitted version.
